# NIR‐II fluorescence imaging with ICG improves intraoperative visualization of pelvic autonomic nerves

**DOI:** 10.1002/ctm2.70602

**Published:** 2026-02-15

**Authors:** Qiaojun Qu, Huilong Nie, Shuang Hou, Xiaoyong Guo, Panxia Deng, Shangqiu Chen, Kunshan He, Zeyu Zhang, Chongwei Chi, Feng Wang, Zhenhua Hu, Jie Tian

**Affiliations:** ^1^ Department of Radiology First Hospital of Shanxi Medical University Taiyuan China; ^2^ CAS Key Laboratory of Molecular Imaging, Beijing Key Laboratory of Molecular Imaging, Institute of Automation, Chinese Academy of Sciences Beijing China; ^3^ Department of Gynecology The Fifth Affiliated Hospital of Sun Yat‐sen University Zhuhai China; ^4^ Key Laboratory of Carcinogenesis and Translational Research (Ministry of Education), Department of Gastrointestinal Cancer Center Ward I, Peking University Cancer Hospital & Institute Beijing China; ^5^ Key Laboratory of Big Data‐Based Precision Medicine of Ministry of Industry and Information Technology, School of Engineering Medicine, Beihang University Beijing China; ^6^ School of Artificial Intelligence, University of Chinese Academy of Sciences Beijing China; ^7^ National Key Laboratory of Kidney Diseases Beijing China; ^8^ Engineering Research Center of Molecular and Neuro Imaging of Ministry of Education, School of Life Science and Technology Xidian University Xi'an China

1

Dear Editor.

Achieving clear identification of pelvic autonomic nerves during hysterectomy continues to pose a major unresolved clinical challenge.^1^ Because these delicate nerves are highly susceptible to injury during parametrial dissection, patients frequently experience postoperative urinary problems that markedly diminish their quality of life.^2^ Consequently, a real‐time imaging technique capable of depicting autonomic nerve fibres at submillimetre resolution is urgently required in clinical practice. Initial observations from a single clinical case indicated that ICG‐assisted NIR‐II fluorescence may offer a promising solution to these visualization difficulties.^3^ Based on this finding, we initiated a prospective pilot investigation to assess whether ICG‐enhanced NIR‐II imaging is safe, feasible, and advantageous for real‐time nerve visualization, and to benchmark its performance against traditional NIR‐I imaging.

We prospectively enrolled ten individuals diagnosed with FIGO stage IB1 to IIA1 cervical cancer in 2022. Inclusion criteria comprised individuals aged between 18 and 75 years, patients with preoperative FIGO stage IB1‐IIA2, absence of urinary system obstruction or hydrops, and normal liver and kidney function. Exclusion criteria included a history of pelvic surgery, receipt of preoperative chemotherapy or radiotherapy, pregnancy and known allergy to contrast media. The characteristics of patients and tumours are summarized in Table S1.

All participants received intravenous ICG (5 mg/kg) 24 h before surgery, a timing selected based on our previous pharmacokinetic studies suggesting optimal neural uptake during this window.^4^ For safety considerations, skin tests were performed prior to ICG administration. During infusion, the ICG solution was protected from light using a black plastic cover. Intraoperative NIR‐II fluorescence imaging was conducted using a customized detection system optimized for the 1000–1700 nm window, using a 792 nm laser for excitation. The imaging distance was fixed at 50 cm. NIR‐I fluorescent imaging was proceeded using DPM‐OPENCAM‐02 imaging system. Both NIR‐II and NIR‐I images were acquired in the operating room under dark conditions, with exposure times ranging from 500 to 2000 ms. The fluorescence images were displayed in real time on a computer monitor placed next to the surgical field.

Pelvic autonomic nerves—including the superior hypogastric plexus (SHP), hypogastric nerves (HN), pelvic splanchnic nerves (PSN) and inferior hypogastric plexus–vesical fibres (IHP‐VF)—were successfully visualized in 9 of 10 patients (90%). The representative images are illustrated in Figure 1A–D. Mean signal‐to‐background ratios (SBRs) were 2.0 ± 0.6 for SHP, 2.0 ± 0.4 for HN, 1.7 ± 0.2 for PSN, and 1.6 ± 0.2 for IHP‐VF, values sufficient for real‐time surgical guidance (Figure 1F). The enhanced penetration depth and reduced scattering of NIR‐II wavelengths enabled the nerves to be visualized with distinct margins even when partially embedded within parametrial tissues.^5^ Notably, NIR‐II delineated bladder efferent branches with diameters as small as 0.47 mm (Figure 1D,E), a level of resolution not achievable by conventional optical modalities.

**FIGURE 1:**
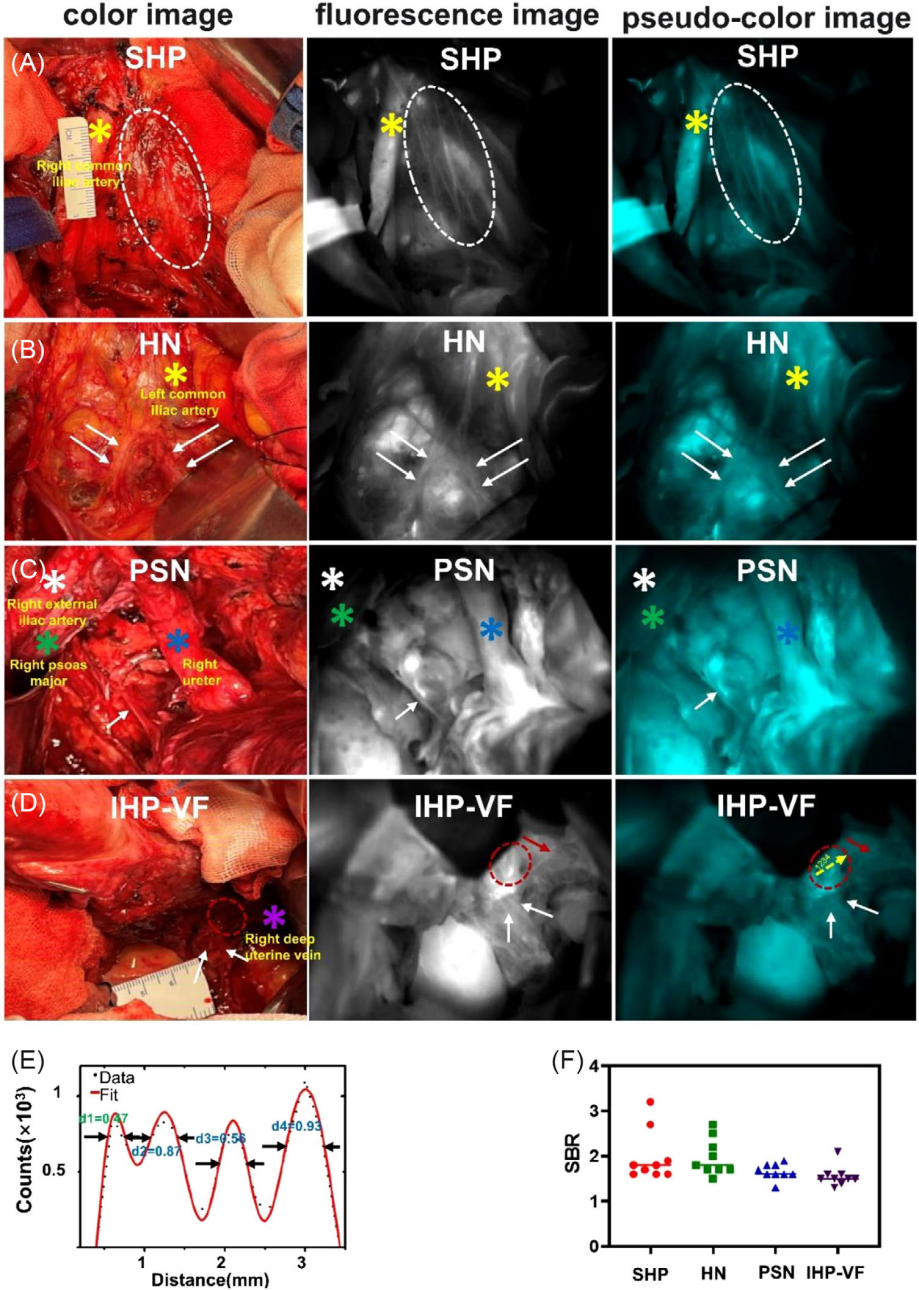
Intraoperative imaging of pelvic autonomic nerves. (A‐D) Colour image, fluorescence image and pseudo‐colour image of SHP (white dashed circle), bilateral HN (double white arrows), PSN (single white arrow), IHP (white arrow)‐vesical fibres (red dashed circle) and‐vaginal fibres (single red arrow); (E) cross‐sectional fluorescence intensity corresponding to the location and direction of the yellow arrow in Figure 1D, which demonstrated the 4 tiny bladder branches could be distinguished from each other; (F) mean SBR of fluorescent pelvic autonomic nerves of nine patients. Yellow star: common iliac artery; white star: right external iliac artery; green star: right psoas major; blue star: right ureter; purple star: right deep uterine vein.

To quantitatively compare the performance of NIR‐II and NIR‐I imaging, three patients underwent simultaneous acquisition using matched fields of view. We examined three representative nerves with varying diameters: the obturator nerve (ON, ∼2.5 mm), the genitocrural nerve (GN, ∼0.8 mm), and the SHP (∼0.5 mm). While ON was detectable using both modalities, NIR‐II provided significantly higher SBR (2.0 ± 0.3 vs 1.5 ± 0.1, *p *= 0.0191, Figure 2A). Remarkably, both GN and SHP were visible only under NIR‐II, with NIR‐I failing to generate any discernible contrast (GN SBR: 1.8 ± 0.3 vs 1.0 ± 0.0, *p *= 0.0062; SHP SBR: 2.2 ± 0.1 vs. 1.0 ± 0.0, *p *< 0.0001, Figure 2B,C). The SBR are shown in Figure 2D. When stratifying nerves by diameter, NIR‐II detected 100% of nerves < 1 mm, whereas NIR‐I detected 0% (Figure 2E). These results demonstrate the unique capacity of NIR‐II to visualize slender pelvic autonomic nerves that are otherwise invisible, filling a long‐standing gap in surgical neuro‐navigation.

**FIGURE 2:**
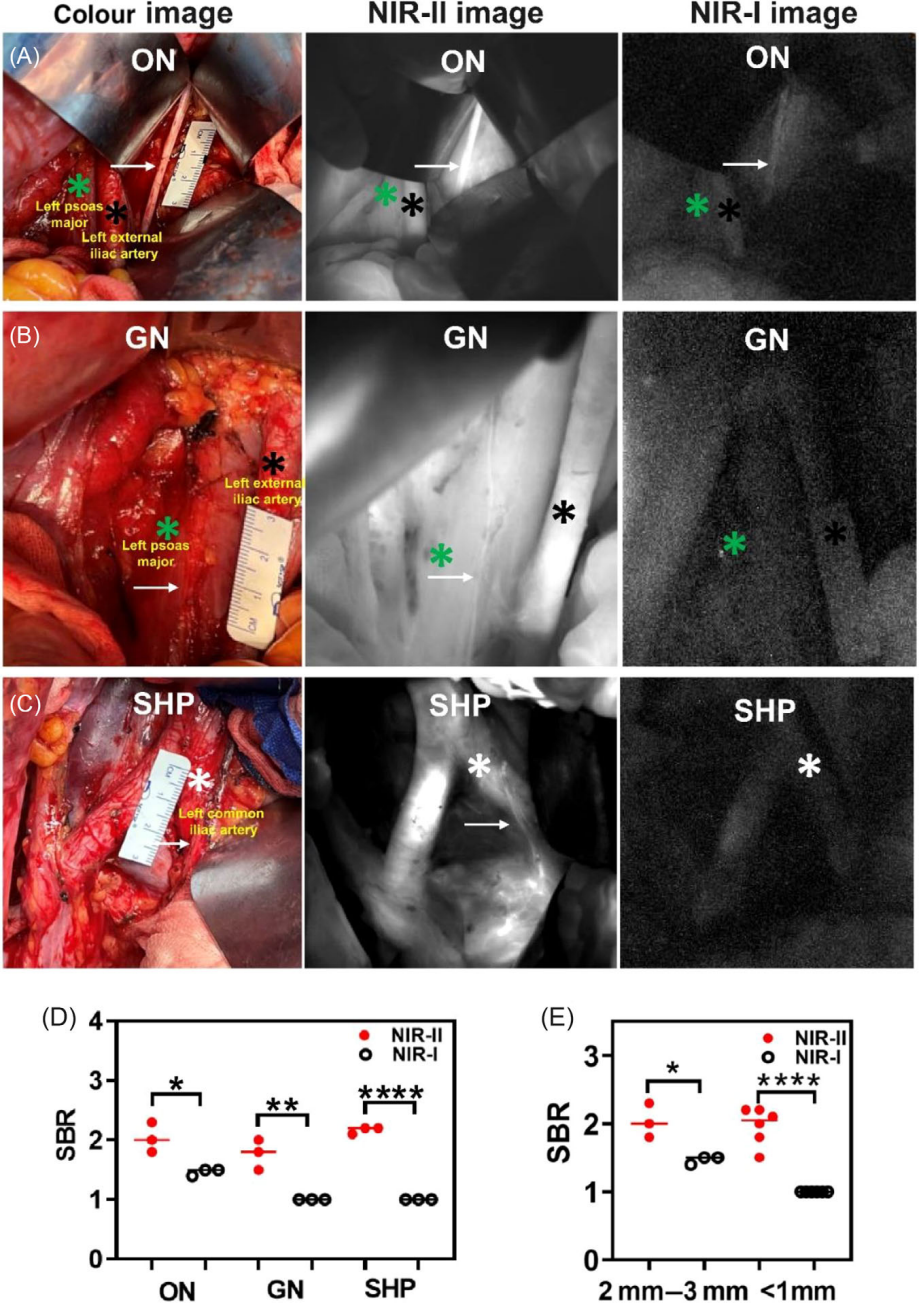
Comparison of the effects of NIR‐II/NIR‐I fluorescence imaging of pelvic nerves. (A‐C) Colour image, NIR‐II image and NIR‐I image of ON, GN and SHP; (D) SBR comparison of different pelvic nerves; (E) SBR comparison of different diameter (2–3 mm vs. < 1 mm) pelvic nerves. Green star: left psoas major; black star: left external iliac artery; white star: left common iliac artery.

Beyond imaging performance, the potential functional benefit of NIR‐II–guided surgery was illustrated in one patient who underwent bilateral parametrial resection, with one side performed conventionally and the contralateral side guided by NIR‐II imaging. Tissue samples from symmetrical regions of the vesico‐cervical ligament were analysed using S‐100 immunohistochemical staining. Quantification revealed that the fluorescence‐guided side had approximately 50% lower nerve density (0.961% vs. 1.922%), suggesting a reduced degree of inadvertent nerve excision (Figure 3A). Functionally, the patient demonstrated rapid postoperative recovery: catheter removal on Day 7 and restoration of normal voiding with a postvoid residual volume of 65 mL (Figure 3B), outperforming typical recovery trajectories reported in patients who underwent nerve‐sparing radical hysterectomy (NSRH), which average around 14 days.^6^ This observation provides early evidence that NIR‐II–guided dissection may promote better preservation of urinary function.

**FIGURE 3:**
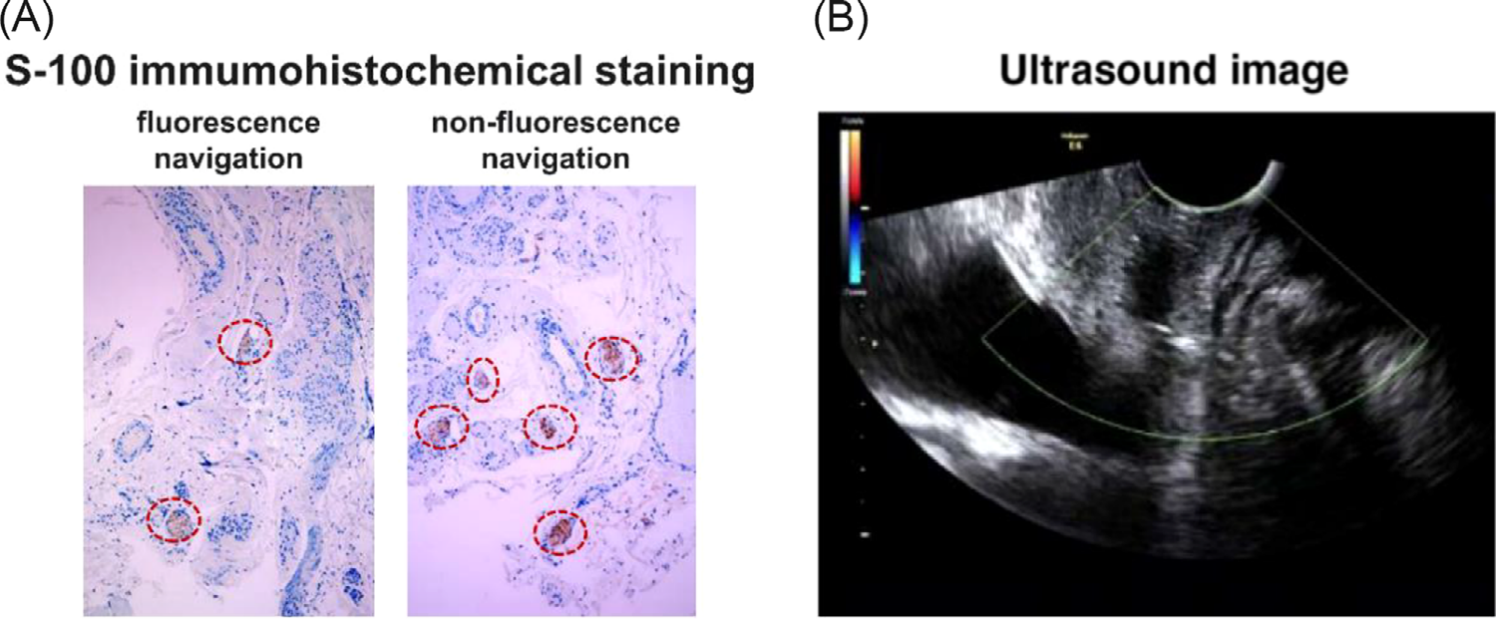
NSRH postoperative evaluation. (A) S‐100 immunohistochemical staining of vesico‐cervical ligaments on the fluorescence navigation side and non‐fluorescence navigation side; (B) bladder ultrasound picture of patient no. 10 on the 7th day after the operation.

To explore the biological basis of nerve‐specific fluorescence, we performed ex vivo microscopic imaging of a pelvic autonomic nerve sample obtained from the uterus specimen. ICG fluorescence strongly colocalized with axonal (TUJ‐1) and myelin sheath (MBP) markers (Figure 4), suggesting that ICG preferentially accumulates within neural structures rather than adjacent connective or vascular tissues. This finding supports the mechanism underlying the favourable nerve‐to‐background contrast observed in vivo.

**FIGURE 4:**
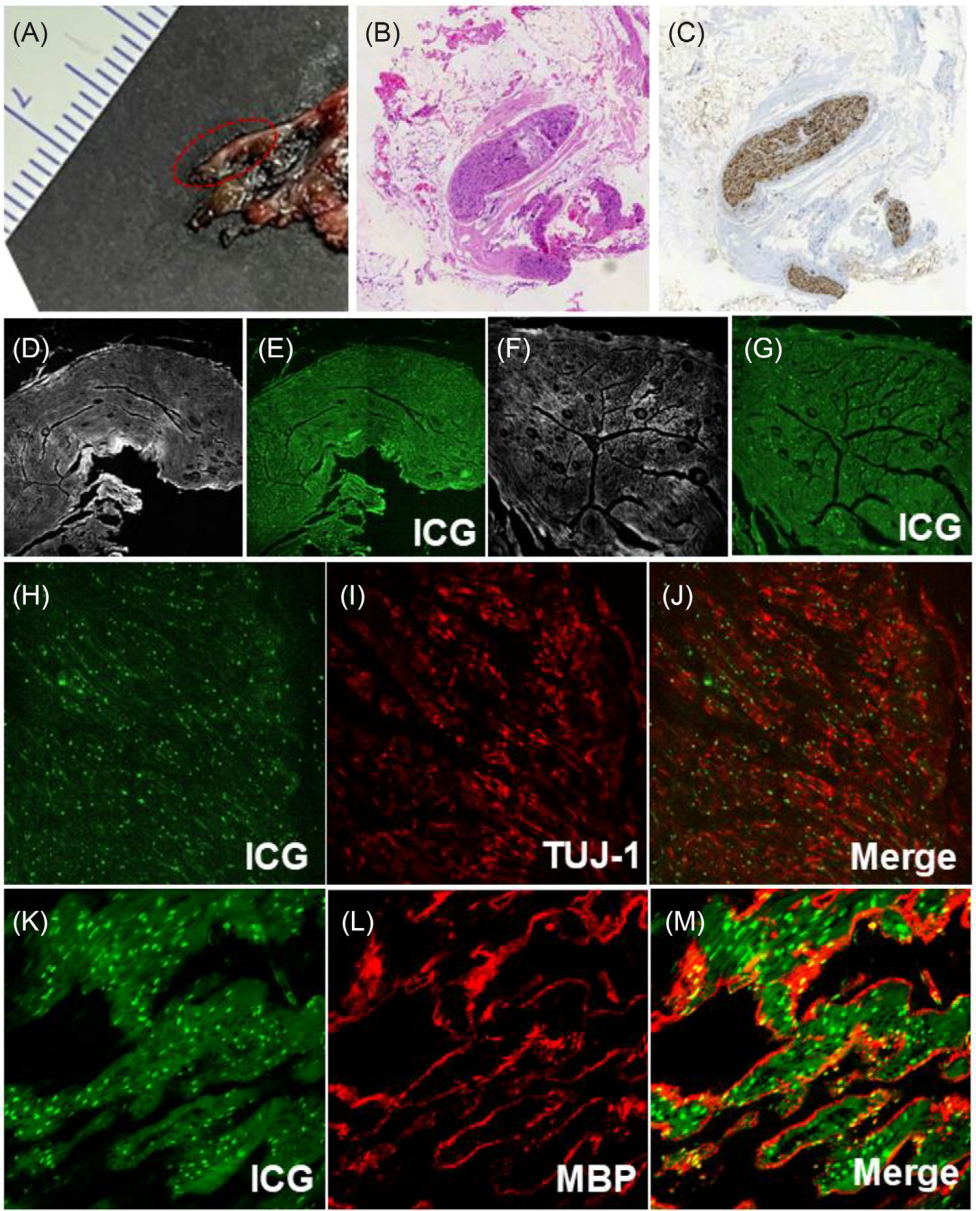
Pathological and microscopic imaging of the pelvic autonomic nerve. (A‐C) Colour image of the pelvic autonomic nerve separated from the isolated uterus, and corresponding HE staining and S‐100 immunohistochemical staining images; (D‐G) microscopic grayscale images (D, F) and ICG fluorescence images of the pelvic autonomic nerve (E, G) (D, E: the magnification was 20×; F, G: the magnification was 40×); (H‐J) different channel microscopic fluorescence images of the pelvic autonomic nerve transverse sections; (K‐M) different channel microscopic fluorescence images of the pelvic autonomic nerve longitudinal sections. (H‐M: the magnification was 40×). Anti‐myelin basic protein (MBP) antibody was used to label the myelin sheath, and TUJ‐1 antibody was used to label axons.

Safety outcomes further support the clinical feasibility of this approach. Only one patient experienced transient postoperative elevation of ALT and AST levels, both returning to normal within two weeks. No allergic reactions, hemodynamic instability, or renal dysfunction related to ICG were observed. The imaging system setup and fluorescence acquisition were smoothly integrated into the standard surgical workflow, indicating strong potential for clinical scalability.

Collectively, our findings demonstrate that NIR‐II fluorescence imaging with ICG enables reliable intraoperative visualization of pelvic autonomic nerves—including fine structures invisible to NIR‐I—and shows strong potential to improve surgical precision and functional outcomes in NSRH. By bridging a long‐standing gap between nerve anatomy and real‐time surgical visualization, this technology represents a meaningful advance in oncologic pelvic surgery. While our findings are promising, the small cohort size (*n* = 10) limits their statistical power and generalizability, particularly for functional outcomes and subgroup analyses. Future large‐sample, randomized and multi‐centre studies are essential to validate the benefits of NIR‐II‐guided surgery and assess its broader applicability. Furthermore, the development of highly specific NIR‐II‐targeted fluorescent probes holds the key to significantly enhancing the efficacy of neural imaging.^7^, ^8^

## AUTHOR CONTRIBUTIONS

Qiaojun Qu, Huilong Nie and Shuang Hou contributed to the data acquisition. Panxia Deng and Shangqiu Chen contributed to the surgical assistance. Xiaoyong Guo contributed to data analyses. Kunshan He, Zeyu Zhang and Chongwei Chi contributed to technical support. This article was written by Qiaojun Qu. Feng Wang, Zhenhua Hu and Jie Tian contributed to the study design, data interpretation and article modification. All authors critically reviewed the manuscript and approved the final version for submission.

## CONFLICT OF INTEREST STATEMENT

The authors declare no conflicts of interest.

## FUNDING INFORMATION

This study was supported by the National Natural Science Foundation of China (NSFC) (grant nos.: 62425116, 82427807, 62027901, 92359301, 92459304 and 81227901), the Sanjin Talent Innovation Team Project of Shanxi Province (grant no.: SJYC2024495) and Shanxi Province Science Foundation for Youths (grant nos.: 202303021212319 and 202203021222374).

## ETHICS STATEMENT

This study received approval from the Committee of the Fifth Affiliated Hospital of Sun Yat‐sen University (approval code: K47‐1) and was registered at ClinicalTrials.gov (NCT05087264).

## PATIENT CONSENT STATEMENT

Informed consent was obtained from the subjects prior to participating in the study.

## Supporting information



Additional supporting information can be found online in the Supporting Information section at the end of this article.

Supporting Information

## Data Availability

Data can be provided upon reasonable request by contacting the corresponding author.
