# Supplementary material for: NIR‐II fluorescence imaging with ICG improves intraoperative visualization of pelvic autonomic nerves
Source: Clin Transl Med. 2026 Feb 15;16(2):e70602. doi: 10.1002/ctm2.70602 (PMC12906991; doi:10.1002/ctm2.70602)
Supplement: Supplementary file 2 — Supporting Information [file CTM2-16-e70602-s002.docx]

**——Supplementary Materials——**

**NIR-II Fluorescence Imaging with ICG Improves Intraoperative Visualization of Pelvic Autonomic Nerves During Radical Hysterectomy**

**Authors**

Qiaojun Qu^1, 2, #^, Huilong Nie^3, #^, Shuang Hou^3, #^, Xiaoyong Guo^4^, Panxia Deng^3^, Shangqiu Chen^3^, Kunshan He^2^, Zeyu Zhang^5^, Chongwei Chi^2^, Feng Wang^3, *^, Zhenhua Hu^2, 6, 7, *^, Jie Tian^2, 5, 6, 7, 8, *^

**Affiliations**

^1^Department of Radiology, First Hospital of Shanxi Medical University, Taiyuan, China

^2^CAS Key Laboratory of Molecular Imaging, Beijing Key Laboratory of Molecular Imaging, Institute of Automation, Chinese Academy of Sciences, Beijing, China

^3^Department of Gynecology, The Fifth Affiliated Hospital of Sun Yat-sen University, Zhuhai, China

^4^Key Laboratory of Carcinogenesis and Translational Research (Ministry of Education), Department of Gastrointestinal Cancer Center, Ward I, Peking University Cancer Hospital & Institute, Beijing, China

^5^Key Laboratory of Big Data-Based Precision Medicine of Ministry of Industry and Information Technology, School of Engineering Medicine, Beihang University, Beijing, China

^6^School of Artificial Intelligence, University of Chinese Academy of Sciences, Beijing, China

^7^National Key Laboratory of Kidney Diseases, Beijing, China

^8^Engineering Research Center of Molecular and Neuro Imaging of Ministry of Education, School of Life Science and Technology, Xidian University, Xi’an, China

**^#^These authors contributed equally to this work.**

**^*^Correspondence to:**

Feng Wang, 1206346068@qq.com

Zhenhua Hu, zhenhua.hu@ia.ac.cn

Jie Tian, jie.tian@ia.ac.cn

| **Table S1. Characteristics of the study patients** | |
| --- | --- |
|  | **Mean±S.D. or N (%)** |
| **Age(years)** | 57.7±10.6 |
| **BMI** | 22.3±2.6 |
| **Preoperative FIGO stage** |  |
| IB1 | 3 (30.0) |
| IB2 | 1 (10.0) |
| IB3 | 1 (10.0) |
| IIA1 | 5 (50.0) |
| **Histologic type** |  |
| adenocarcinoma | 2 (20.0) |
| squamous cell carcinoma | 8 (80.0) |
| **Cervical conization** |  |
| yes | 2 (20.0) |
| no | 8 (80.0) |
| BMI, body mass index; FIGO, International Federation of Gynecology and Obstetrics; S.D., standard deviation | |
